# Understanding Barriers to Home Safety Assessment Adoption in Older Adults: Qualitative Human-Centered Design Study

**DOI:** 10.2196/66854

**Published:** 2025-06-24

**Authors:** Jonathan J Lee, Devika Patel, Meghana Gadgil, Simone Langness, Christiana Dagmar von Hippel, Amanda Sammann

**Affiliations:** 1 School of Medicine, Stanford University, Stanford, CA, United States; 2 Hasso Plattner Institute of Design, Stanford University, Stanford, CA, United States; 3 The Better Lab, University of California San Francisco, 2540 23rd St, 4th Floor, San Francisco, CA, 94110, United States; 4 Department of Surgery, University of California San Francisco, San Francisco, CA, United States; 5 Department of Medicine, University of California San Francisco, San Francisco, CA, United States; 6 Sharp HealthCare, San Diego, CA, United States

**Keywords:** human-centered design, fall prevention, home safety assessments, occupational therapy, aging in place, elderly, older adult, design, fall, home safety, safety, therapist, aging, perspectives, community-dwelling, development, qualitative research, qualitative data, semi-structured interviews, interview, occupational therapists, geriatricians, surgeons

## Abstract

**Background:**

Falls are the leading cause of injury-related death among adults aged 65 and older. The fear of falling can further limit older adults’ independence by contributing to activity restriction, social isolation, and physical decline—ironically increasing the risk of mechanical falls. Although home safety assessments have been shown to reduce fall risk by up to 36% and decrease serious injuries such as hip fractures, their adoption remains low. Understanding the barriers to implementing these assessments is critical to improving their uptake and effectiveness.

**Objective:**

This study aimed to (1) identify specific barriers perceived by older adults in implementing home safety assessments and modifications to reduce the risk of mechanical falls, (2) explore the attitudes of health care professionals and other stakeholders toward these assessments, and (3) identify novel design opportunities to guide the development and implementation of more effective home safety assessment techniques and practices to reduce mechanical fall risk.

**Methods:**

This explanatory qualitative study drew on the “inspiration” phase of the human-centered design (HCD) research process. We conducted 35 interviews (28 initial and 7 follow-up) with 28 purposefully sampled participants in the San Francisco Bay Area between February and June 2021. Participants included community-dwelling older adults (n=3), geriatricians (n=4), therapists (n=6), product developers (n=2), older health researchers (n=8), and community program leaders (n=5). Interview notes were analyzed inductively by the research team to extract themes and generate insight statements and design opportunities.

**Results:**

Analysis yielded three key insights: (1) older adults often experience a conflict between maintaining independence and implementing safety modifications. One participant described living with a “repeating mantra in my head throughout the day saying ‘above all, don’t fall.’” (2) aesthetic and privacy concerns frequently override safety benefits. Participants rejected modifications that made their homes feel “institutional.” (3) access to occupational therapy services—already limited in rural areas—was further constrained by the COVID-19 pandemic, with some providers reporting that travel time “took up the majority of their day just assessing one home.” These barriers help explain the low adoption of home safety assessments despite strong supporting evidence. The study identified design opportunities to address these challenges, including customizable, user-friendly safety solutions, dignity-preserving approaches to assessment, and technology-enabled remote alternatives.

**Conclusions:**

This study identified specific emotional, aesthetic, logistical, and access-related barriers to the adoption of home safety assessments among older adults. The proposed design solutions offer promising directions to increase uptake, improve user experience, and enhance safety. However, further validation through co-design with a larger and more diverse group of older adults is needed. Future research should pilot test these ideas across varied contexts and evaluate their implementation and impact.

## Introduction

A total of 1 out of 3 people aged 65 years and older falls annually, and falls are subsequently the leading cause of injury-related death in this age group [1,2]. Falls result in 2.8 million emergency department visits every year, with 25% of admissions being associated with serious injuries, such as fractures or traumatic brain injury, which can permanently decrease quality of life [3]. Fatal and nonfatal falls cost an estimated US $50 billion in the United States in 2015, which increased up to US $80 billion in 2020, with the bulk of the cost attributed to nonfatal falls and paid for by Medicare [4,5].

Falls and fear of falling in turn threaten older adults’ ability to live independently and often lead to self-imposed activity restriction, social isolation, decreased cognitive and physical function, and, paradoxically, an increased risk of serious falls [6,7]. The emotional impact of falling is grave, with studies finding that 80% of older women would prefer death to a bad hip fracture that would lead to nursing home admission [8]. Alternative solutions to receiving 24-hour monitoring and high-level medical care in a nursing home, such as moving into an assisted living community where support is available for activities of daily living, are a last resort for many. The vast majority (90%) of older adults aged 50 years or older prefer to age in place in their current home [9]. However, 85% of these same individuals who desire to remain living at home have not adequately prepared their homes for aging in place through efforts such as removing trip hazards and installing assistive devices (eg, bathroom grab bars) [9]. Finding a wide range of ways to reduce falls can potentially both increase the quality of life for older adults choosing to age independently and also lead to large savings in direct medical costs [10].

The risk of falling and resulting serious injury are associated with many modifiable and nonmodifiable risk factors. Nonmodifiable risk factors include age, cognitive impairment, female sex, and a history of falling [11]. Modifiable risk factors include balance impairment, gait impairment, muscle weakness, medication use, and home environment [11]. Unfortunately, there is no one single risk factor for falls upon which to intervene. Fall risk is multifactorial, and falls tend to occur when impairments exist in multiple domains of an individual’s health [12].

There has been extensive research on interventions that can reduce the risk of fall-related injury and mortality in the older adults. For example, home safety assessments performed by an occupational therapist for community-dwelling older adults have been shown to significantly decrease the rate and risk of falling by up to 36%, along with reducing serious fall-related complications, including hip fractures and death [13,14]. Keall et al [15] randomized 842 senior households to an intervention involving a standardized set of home safety modifications including staircase handrails, bathroom grab bars, improved lighting, nonslip mats and rugs, and an educational brochure on home safety, which demonstrated a 26% decrease in fall injuries in the intervention group compared with the control group.

Despite the clear, data-driven benefits of home safety assessments and related home environmental improvements, adoption of these services remains low for a variety of reasons. Some insurance companies that previously covered home safety assessments as a benefit for their members have discontinued this benefit due to low use by older adult policyholders [14,16]. One key reason for the underuse of home safety assessments is that many older adults perceive a visit from a health care provider as a reminder of their inevitable aging and an invasion of their privacy, with some reacting to suggested changes as an encroachment on their personal independence, dignity, and sense of control [17-19] Personalized care approaches that prioritize individual preferences can significantly improve intervention acceptance and perceived autonomy [20]. Furthermore, concerns about loss of independence often outweighs the fear of injury, leading older adults to underuse fall prevention interventions [21].

Older adults’ resistance to addressing the physical, social, and emotional changes associated with aging may be exacerbated by their minimal inclusion in decision-making processes, particularly those related to their living spaces [18,22]. Multiple studies suggest that this exclusion increases resistance to home modifications and other aging-related changes, as older adults often feel a loss of control when they are not actively involved in decisions affecting their environment [19,20]. By involving older adults in these decisions, their sense of autonomy and dignity can be maintained, which in turn reduces emotional distress and increases acceptance of necessary changes [21].

Understanding the barriers older adults face in using home safety assessments is critical to addressing modifiable fall risk factors and reducing falls. This study aimed to (1) identify specific barriers perceived by community-dwelling older adults in accessing and implementing home safety assessments and modifications, (2) explore attitudes of health care professionals and additional stakeholders toward home safety assessments, and (3) identify novel design opportunities to guide the development and implementation of more effective home safety assessment techniques and practices.

## Methods

### Study Design and Setting

This explanatory qualitative research study used human-centered design (HCD) methodology to understand the barriers to home safety assessments and develop opportunities to support fall prevention for community-dwelling older adults [23,24]. The study was performed as a collaboration between Stanford University’s Design for Extreme Affordability course within the Hasso Plattner Institute of Design (d.school) and The Better Lab, an academic research laboratory focused on HCD in health care, based in the Department of Surgery at the University of California, San Francisco. Study activities took place between February and June 2021, with interviews conducted both virtually and in person at Stanford Hospital and Clinics.

### Research Team Characteristics

The research team consisted of 3 designers from Stanford’s d.school, 2 clinical researchers from the University of California, San Francisco (both practicing geriatricians), and 2 HCD researchers from “The Better Lab.” Team members had expertise in geriatric medicine, occupational therapy, design research, and public health. A total of 4 members had prior experience conducting qualitative research with older adults, and all researchers received training in qualitative interviewing techniques to ensure consistency. We acknowledge that our clinical and design backgrounds may have influenced our focus on intervention development. To mitigate bias, our team engaged in regular discussions to examine assumptions and incorporate peer debriefing throughout the study. None of the researchers had prior relationships with study participants.

### Participant Selection

We used purposeful sampling to ensure heterogeneity and diverse representation of stakeholder perspectives [25]. Our sampling strategy aimed to include individuals with varied relationships to home safety assessment implementation, including (1) community-dwelling older adults who might benefit from assessments, (2) health care providers who conduct or refer for assessments, (3) family members who support older adults in making home modifications, (4) program administrators who oversee assessment services, (5) product developers working on related technologies such as assistive technology, smart home solutions, or fall prevention tools.

Our selection criteria included age (65+ years for older adults), direct involvement in home safety assessments, and willingness to discuss their experiences. We specifically sought older adults with varying experiences with falls and home modifications, ranging from those who had never had an assessment to those who had implemented modifications. Health care providers were selected to represent different specialties and practice settings.

### HCD Process

Over the past decade, the use of HCD in health care has grown substantially. Case studies and qualitative analyses suggest that using HCD approaches may enhance patient engagement and improve service delivery, potentially leading to better health care access and outcomes [26-28]. HCD is problem-solving methodology rooted in engineering, psychology, anthropology, business, and design that brings together designers and stakeholders of an issue to identify challenges and use an iterative process to cocreate solutions [29]. HCD uses ethnographic research methods to identify unmet needs and contextualizes the study challenge within the greater ecosystem to identify impactful and sustainable solutions [24,26-28].

The HCD process consists of three phases: inspiration, ideation, and implementation [30]. The inspiration phase involves identifying unmet needs through interviews and observations with key users and related stakeholders [31]. Qualitative data from these interviews and observations are synthesized to identify themes and inform *t*he creation of insight statements and design opportunities. The ideation phase involves convening users, stakeholders, and designers for group workshops aimed at brainstorming potential solutions and co-designing prototypes of the most desirable, feasible, and viable potential solutions [30,32]. Finally, the implementation phase consists of iterative testing, incorporating user and stakeholder feedback to refine prototypes for pilot testing [30]. This paper describes the inspiration phase of our study in which we explored barriers to home safety assessments and modifications by interviewing older adults, health care professionals, and other key stakeholders.

### Inspiration Phase Qualitative Data Collection and Analysis

We recruited all participants using purposeful sampling to ensure heterogeneity and a diverse representation of perspectives in the study cohort, consistent with the HCD methodology [33]. Recruitment criteria involved individuals directly involved with home safety assessments or fall prevention. We obtained qualitative data through interviews with older adult users and other key stakeholders, including clinicians (physical therapists, occupational therapists, geriatricians, and surgeons), family members of older adults, engineers, senior living community administrators, interior designers, and payers. We created a semistructured interview guide to understand factors that contribute to falls, experiences within the health care system following a fall, and the psychological impact of falls.

We interviewed a total of 28 participants for 30‐60 minute interviews conducted virtually over Zoom (Zoom Communications, Inc) or in person at Stanford Hospitals and Clinics between February and June 2021. In light of COVID-19 restrictions, we conducted interviews both virtually and in-person, and in both settings we ensured participant comfort and openness to create a flexible environment for discussion. Two to three researchers per session conducted the interviews, with 1 lead researcher leading the interview while the other researchers transcribed detailed notes.

**Figure 1.**
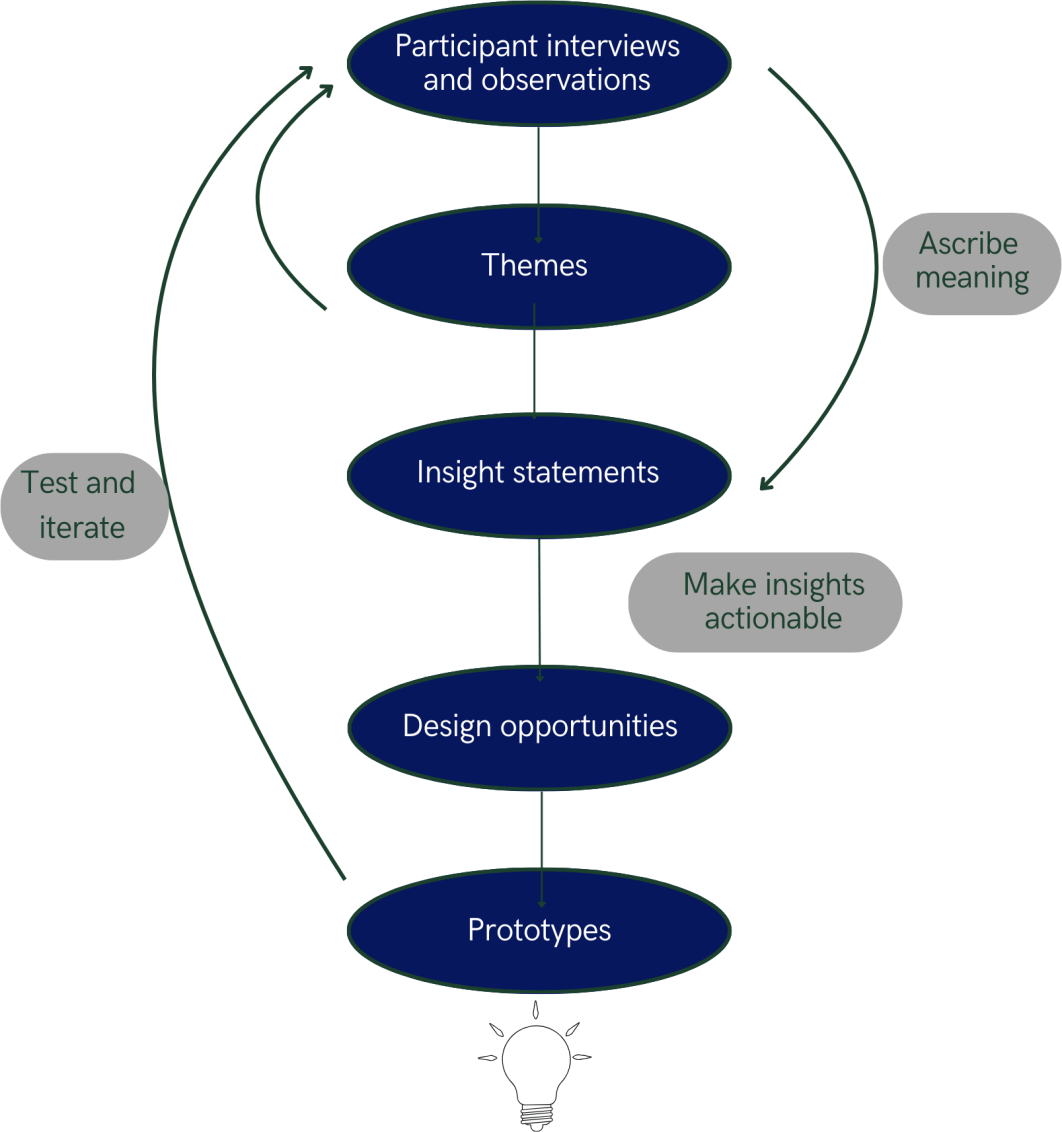
Human-centered design qualitative data analysis process diagram.

Figure 1 illustrates this study’s multistep HCD qualitative analysis process, which we followed up to the step of developing design opportunities. We followed a 3-step approach using reflexive thematic analysis [34]. First, 3 team members, who were students with a background in design and working in partnership with a HCD expert, independently conducted an inductive thematic analysis of the interview notes to identify emerging themes and refined them through discussions until consensus was reached [35,36]. Second, the entire research team collaboratively developed and reached consensus on multiple insight statements, capturing relational themes such as tensions, synergies, barriers, and facilitators to health behavior described by participants. Third, all research team members collaborated to translate each insight statement into 1 or more design opportunities through structured discussions, with each opportunity representing actionable concepts or intervention elements that directly address the challenges identified in participant responses [37],[38-39].

### Ethical Considerations

This study was determined to be exempt from review by Stanford University’s Institutional Review Board (60770). All participants provided verbal informed consent after reviewing study information. Given the sensitive nature of discussions about falls and home modifications, we took particular care to allow participants to skip any uncomfortable topics, provide support resources when appropriate, and to protect confidentiality in our data management. Participation was voluntary, and interviewees received US $20 gift cards as compensation. Interviews were anonymized to protect patient confidentiality. No interviews were audio or video recorded due to the sensitive and confidential nature of these conversations.

## Results

### Overview

A total of 35 interviews (28 initial and 7 follow-up) were conducted between February and June 2021. Our participant group comprised 28 diverse stakeholders: 3 community-dwelling older adults, 4 geriatricians, 6 therapists (including physical, occupational, and mental health–focused practitioners), 2 product managers, 8 researchers specializing in older health, and 5 leaders of older care programs and communities. Of these, 18 (64%) participants identified as women. Interview lasted between 30 and 60 minutes.

Through inductive analysis of interview data, we identified 3 key insights regarding the interconnected and sometimes opposing perspectives of older adult users, occupational therapists, community living organizations, and geriatricians on fall prevention and home assessments. Below, we present these insights along with proposed design opportunities that emerged from our analysis.

### Insight 1

Older adults have a strong desire to maintain active, independent lifestyles that align with a sense of youthfulness, yet alterations to their environments and lifestyles that could enhance their well-being and safety are perceived as undermining independence, vitality, and creating fear around being active.

Older adults may be overwhelmed and disoriented by an increasing loss of control over their health, lifestyle, and relationships. For example, one older adult interviewee described an all-consuming fear of falling following her first fall, such that she hears a repeating mantra in her head throughout the day saying, “above all, don’t fall.” Much like an initial fall, a visit from a health care provider can remind older adults about their inevitable aging, which may contribute to resistance against home assessments. One geriatrician interviewee observed that “patients are reminded of their mortality when an occupational therapist comes to the home to visit.” In addition, multiple stakeholders shared the perspective that during home assessments there is often an extensive list of home modifications that are suggested to make older adults’ homes safer without much consideration of how older adults feel about these changes. For example, one older adult user who had grab bars placed throughout her home reflected that “the way they looked around her home” was a key reason she did not want them installed. The gray color and material of the standard grab bar reminded her of the hospital room, she stayed in for treatment of a former injury, which she wanted to avoid recreating in her home. This suggests that to increase adoption of an effective fall-prevention or safety assessment service, users need to feel motivated and not fearful to make changes to their lifestyle and that engaging users in customizing adaptations to their aesthetic preferences may be a method to improve users’ perception of control.

Much like baby-proofing a home is a sign of natural change in a family unit to protect their well-being, “age-proofing” serves a similar purpose for older adults. As one gerontology expert interviewee noted, “Homes age over time as well, and there’s this idea that a home ages and changes with you.” However, choosing not to “age-proof” their home may be an older adult’s way of rejecting the notion that their lifespan is limited. For example, older adults in our interviews have expressed sentiments such as, “We don’t want to go into a nursing home or senior housing because that’s where old people live.” This fear of aging may lead to a maladaptive way of dealing with the aging process in some individuals, similar to how a fear of falling can paradoxically increase the risk of falls.

#### Design Opportunity 1

Return a sense of control and autonomy to older adults in the home redesign or adaptation process. A home safety assessment tool can be designed to prioritize customizability and facilitate direct feedback from users. The essential features of this product include:

##### Active Participation

Engaging older adults as active participants and decision-makers in any home redesign or change so that they can shape their own personal aging experience, which is currently often overlooked.

##### Customizable Solutions

Providing a customizable system of home modifications that older adults can use to experiment with different materials or patterns (eg, mahogany wood for safety devices such as grab bars) to discern their aesthetic and functional preferences.

##### Positive Framing

Generating enthusiasm from older adults for home modifications by framing them as enhancing rather than diminishing independence, helping them feel more aligned in their goals of care with occupational therapists and geriatricians.

##### Growth Mindset Toward Aging

Encouraging a growth mindset to aging that emphasizes openness to adapting in a healthy, safe, and dignified manner while maximizing the opportunities available to older adults for engaging in daily life activities and aging in place.

### Insight 2

Older adults are willing to risk their safety, even their lives and health, to protect the familiarity of their homes from the change and invasion of privacy associated with home safety assessments.

Older adults know the layout of their homes intimately; one older adult interviewee described this as follows: “We know our home just as well as you know the layout of your childhood home… and know how to avoid the tiny things even when waking up in the middle of the night.” An academic public health specialist in California who focuses on issues affecting the geriatric population, expressed in an interview that “[Older adults] are reluctant to let a stranger into their homes to change things.” Multiple older adult interviewees expressed that they had already heard from their children and relatives that they need to make their home safer through modifications but had rejected that advice. One older interviewee cited aesthetics as the reason for rejecting suggested changes saying, “My kids have been telling me for months now to clean up my clutter and install a grab bar in my shower, but I just don’t like the way it looks in my home.” However, according to a geriatrician interviewee, when considering accepting support to adapt their homes to support aging in place, “the elderly struggle with dignity.” Home was described by multiple older adult interviewees as representing a personal haven, or area of refuge and constancy away from the influences and preferences of others. Maintaining control over their home may be considered an aspect of living with dignity for the community-dwelling older adults in the current sample.

Both older adult users and clinicians shared in interviews that resistance to changing the home persists even after an older adult’s first fall, suggesting that users’ disinterest in making home modifications is not rooted in concern for their health, but rather in the emotions associated with aging, change, and privacy. This idea is supported by the perspective of a fall prevention center leader who stated in their interview that “They [older adults] don’t want to change, and this term [home modifications] isn’t enough to conquer their fear of change.” The design opportunities that we framed from the insights gleaned from interview data, therefore emphasized understanding and addressing older adults’ powerful emotions and feelings of fear of change and loss of independence in any solution.

#### Design Opportunity 2

Reframe how home safety assessments are perceived by users so that older adults can become fully engaged in the broader process of safely aging in place while protecting themselves and preserving their dignity and independence. The home safety modification process can become an engaging opportunity for an older adult to design their personalized “forever” home while also enhancing their safety. The essential features include:

##### Inspiring a Positive Outlook

Encouraging older adults to embrace the next stage of their life using home safety assessments and modifications.

##### Connecting With Trusted Professionals

Linking users with local contractors who can install both the necessary fall prevention devices as well as any additional renovations that the older adult would like to enhance their safety while maintaining the comfort of home.

##### Providing Educational Resources

Offering supplemental educational resources about changes that occur during aging to help users conquer their fear of losing dignity and independence.

### Insight 3

Access to home safety assessments would improve older adults’ safety yet they are perceived as unsafe or inaccessible, especially during the COVID-19 pandemic. The COVID-19 pandemic illuminated and exacerbated the existing misalignment in supply and demand for occupational therapists and home safety assessments. It also opened opportunities to assess technological approaches to work on the problems of accessibility and acceptability of home safety assessments for older adults. Many community and aging organizations such as Ashby Village in California, have turned to trained volunteers to perform home safety assessments for individuals within their network given the high demand for occupational therapists. During the COVID-19 pandemic, when quarantines and social distancing were established, in-person home safety assessments within these communities were halted. During our interview in 2022, Ashby Village organizers mentioned to us that “we haven’t done any home assessments since March 2020 and do not plan on doing any for the foreseeable future.” Though they resumed this service postpandemic, in the interim many users who might benefit from a low-cost fall prevention service were left without access. Even though larger health care networks continued to perform in-person visits during the pandemic, we heard from older adult users about their resistance to having a stranger come into their home. “If I can’t even feel safe inviting family into my home, why would I invite a complete stranger in?”

Finally, even large health care networks have struggled to reach older adults who live in rural areas. As one interviewee noted, a home safety assessment process for such an individual would consist of “sending an occupational therapist on a 2-hour commute each way to see this person which can take up the majority of their day just assessing one home.” This suggests a current unmet need in the home safety assessment space, which likely disproportionately affects rural and underserved populations and will continue to grow as the population ages.

#### Design Opportunity 3

Increase access to home safety assessment tools using alternative and innovative technologies. Online home safety assessment tools would allow users to benefit from a home safety assessment and a reduced fall risk, even with fewer volunteers and limited in-person visits due to the pandemic. The essential features of such a tool could include:

##### Online Home Assessments

Developing an online assessment tool which allows users to submit photos of various rooms in their home for review by licensed occupational therapists, which can produce a risk score for each room and identify areas that should be addressed.

##### Future Adaptability

Implementing computer vision and machine learning, which can examine visual data representations and automate the home safety assessment process over time.

##### Personalized Recommendations

Combining visual data with user surveys that contain their demographic information and design preferences to generate personalized home modification suggestions, ensuring adaptations accommodate mobility aids (eg, walkers), while remaining functional and aesthetically pleasing to the user.

Further possibilities using a wide range of technological tools and equipments, including smartphone and tablet apps, video games, wearable devices, and future robotic efforts to improve home safety assessments. Their potential impacts on fall prevention efforts are discussed in the “Discussion” section.

## Discussion

### Principal Findings

This study provides insights into the complex factors affecting the adoption of home safety assessments among older adults and suggests potential directions for improving implementation. Our findings highlight 3 key areas of tension existing between stakeholders in aging in place process, including older adults, clinicians, home assessors, and others: maintaining independence while ensuring safety, preserving home sanctuary while accepting modifications, and balancing assessment access with implementation constraints. Since older adults in the current sample desired to preserve their independence and dignity, they were resistant to implementing recommended changes, especially those related to their home safety, as they wanted to avoid the negative emotions associated with considering how aging impacts physical and other abilities. The strong emotional responses to home modifications revealed in our study align with previous research showing that older adults often perceive environmental changes as threats to independence [40]. However, our findings extend this understanding by highlighting how aesthetic and personal meaning considerations may override known safety benefits. This suggests that purely educational or risk-focused approaches may be insufficient to drive behavior change.

The COVID-19 pandemic’s impact on assessment delivery highlighted both challenges and opportunities in service provision. While disrupting traditional in-person assessments, it accelerated exploration of remote alternatives. However, technological solutions must carefully consider older adults’ preferences and potential barriers to adoption.

Technology has become increasingly integrated into the fall prevention research and health care field, especially following the 2008 decision by the Centers for Medicare and Medicaid Services to stop reimbursing costs related to in-hospital falls [41,42]. For example, smartphone and tablet apps have been developed to encourage greater levels of exercise in older adults, which in turn can reduce falls [43]. Similarly, video games such as the Wii Fit have been successfully used to perform mobility-based fall risk assessments in older adults [44]. Wearable sensors have also been increasingly studied, with a systematic review identifying 40 studies and 130 distinct variables examining sensor use in geriatric falls with promising results [45]. Technologically supported interventions may reduce fall risk either by directly minimizing an older adult’s physical risk of falling or by improving their confidence to reduce their fear of falling, which is an important predictor of falls. Older adults’ adoption of wearable sensors such as fitness trackers could lead to the accumulation of ”big data”—a large-scale dataset automatically collected from the sensors themselves, potentially enabling analysis and detection of patterns in fall risk and incidence to be identified more easily—though, this would depend on greater uptake of digital technologies in this population than has yet occurred.

Only a limited number of studies have used technology specific to the home safety assessment process. For example, Du et al [46] used a robotic system operated remotely by occupational therapists to conduct home safety assessments. In addition to providing a video livestream of the home, the system was able to measure distances between objects, detect lighting situations, and communicate with the older adult. Furthermore, ambient camera-based sensors have also been tested for automatic fall detection [47]. However, both solutions face challenges in uptake, given the cost associated with the technological systems and the time needed for installation and for the user to learn the system. Another study collated a protocol to be used for remote home safety assessments through extensive secondary research and interviews with occupational therapists and caregivers [48]. Taken together, these previous studies and our current one highlight the increasing recognition of home safety assessments as a potential area of improvement in fall prevention, and evidence of the value of future investments in user-friendly technological interventions for fall prevention and awareness among older adults.

### Limitations

This study has several important limitations. First, while we sought to understand older adults’ perspectives, only 3 of our 28 participants were older adults themselves. This occurred primarily due to recruitment challenges related to COVID-19. This limited representation may not capture the full range of older adult views and experiences. Future work should more deeply engage older adults both as study participants and as co-designers of proposed solutions. Second, our proposed design opportunities emerged from researcher analysis rather than cocreation with stakeholders. While grounded in participant insights, these proposals require validation through future participatory design work with end users and stakeholders. Third, all participants were recruited from the San Francisco Bay Area, potentially limiting generalizability to other geographic and cultural contexts. Health care access, technology adoption, and attitudes toward aging may differ substantially in other regions. Finally, our use of interview notes rather than full transcripts may have missed subtle themes or nuances in participant responses. This approach was selected to maintain the confidentiality of participants experiencing a sensitive issue. Future studies would benefit from recorded and transcribed interviews to enable more detailed analysis.

### Conclusions

This exploratory study reveals important considerations for improving home safety assessment implementation. Successful interventions likely require careful attention to older adults’ emotional needs, aesthetic preferences, and desire for autonomy. While technology-enabled approaches show promise for expanding access, they must be developed in partnership with older adults to ensure acceptability and adoption. Our findings suggest that addressing these factors could potentially lead to more effective interventions and increased adherence to home modifications.

Future research should involve larger, more diverse samples of older adults to validate and refine proposed design opportunities using co-design methods. Prototypes of promising approaches should be pilot tested in varied geographic and cultural contexts. It will be important to then evaluate both implementation outcomes and impacts of pilot-tested interventions on fall prevention.

In addition to research advancements, our findings have practical implications for clinical practice. Training programs for occupational therapists should incorporate strategies to address older adults’ emotional and psychological barriers to home modifications. Furthermore, the development of educational materials tailored to older adults’ concerns—such as maintaining dignity and control while making safety modifications—could facilitate better engagement with home safety interventions.

Rather than prescribing specific solutions, this work provides a foundation for human-centered approaches to fall prevention that honor older adults’ dignity while promoting their safety. By integrating these insights into practice, health care professionals and designers can create more effective, personalized, and widely adopted fall prevention strategies.

## Abbreviations

HCD: human-centered design
